# Numerical Investigations of the Seam Opening Behavior of Peelable Seal Seams as a Function of the Seal Seam Formation

**DOI:** 10.3390/polym17172407

**Published:** 2025-09-04

**Authors:** Marc Götz, Fabian Kayatz, Marek Hauptmann

**Affiliations:** Fraunhofer Institute for Process Engineering and Packaging IVV, Division Processing Technology, 01189 Dresden, Germany

**Keywords:** heat contact sealing, T-peel test, seal seam formation, seal opening behavior, FEM, LS-DYNA, cohesive elements

## Abstract

In the process of heat contact sealing of thin, flexible polymer films, the choice of the film material, the layer structure, the sealing tools, and the process parameters influence the melt flow. A pronounced melt flow dynamic, which is to be expected in industrial applications due to the high temperatures and pressures, favors the formation of sealing edges that negatively affect the user-friendliness when opening the packaging. In this study, the influence of seal seam formation on the opening behavior was systematically investigated by numerical simulation. A detailed model was developed that simulates the seam opening process, accounting for the composite structure, the detailed geometry of the seal seam, and the separation process. The numerical results of the force-offset behavior showed good agreement with experimental data. Parameter studies revealed that the thicker and more pronounced seal seams lead to higher tear-off forces, while thinner and less pronounced seams result in lower forces. These findings provide valuable insights into the interactions between seam formation and the mechanical behavior of flexible films, enabling the optimization of sealing processes for improved package performance.

## 1. Introduction

Heat contact sealing is the most commonly used process in the packaging industry to join polymer films [1]. In this technology, two films are pressed together by two movable heated seal jaws. Heat transfer from the heated sealing jaws through the film is established to achieve entanglement at the interface between the films. In industrial applications, high sealing forces are necessary to compensate for layer jumps and side creases and to fill these with sufficient melt, as well as to bridge possible contamination in the sealing seam with the packaged goods and, thus, ensure safe packaging.

However, practice-relevant sealing forces result in pronounced melt flow, which leads to different seal seam formations, which in turn influence the opening behavior of the sealing seam as shown in scientific papers such as [2,3,4]. The sealing seam is significantly influenced by the sealing method, such as heat contact sealing, ultrasonic sealing, or induction; the process parameters; and the geometry used [5]. However, the formation of the sealing seam is critical. On the one hand, these prevent sufficient adhesion in the near-edge area, and on the other hand, they have a negative influence on the peelability due to the displacement of the peel layer. As a result, peelable films may lose their peelability. Ref. [6] describes a model for calculating the melt flow. However, it is assumed that the medium exhibits Newtonian behavior. Calculations show that the melt flow increases linearly with pressure and time, and quadratically with increasing temperature. An investigation of the seal seam formation and its effects on the seam opening behavior was not carried out. Other scientific publications on heat contact sealing are limited to the investigations without pronounced melt flow, as the sealing pressure is assumed to be very low, and therefore, not relevant [6,7,8]. Although [2] reports higher seal seam strengths for higher sealing forces, the correlations and reasons were not investigated.

The central quality test for sealed packaging is the T-peel test according to DIN 55529 or ASTM D 1876 [9,10]. In the case of peelable seams, fracture type C usually occurs, i.e., a cohesive fracture at the interface of the sealed films. However, investigations show that even with break type C, different characteristics occur during peeling, which can range from a simple peel effect to significant increases in the force at the start of the test, to the delayed onset of the peel effect due to previous strong elongation of the peel arm. In [4], it has already been established for seal seam geometries from ultrasonic sealing that the seam geometry had a significant influence on the measured force–displacement curve in the T-peel test. However, the seal seam formation cannot be considered as an influencing factor in the T-peel test due to its microscopic size.

Numerical simulation programs offer the possibility of mapping complex geometries and processes on smaller scales that would be difficult to achieve experimentally. In [4], the microscopic formation of the sealing bead of permanently sealed seams is digitized and meshed for the first time, and the seam opening process is simulated, but only up to the crack initiation. The local stress states could be simulated, and the crack progression along the morphological boundary could be estimated in the experiment. Ref. [11] describes an approach to simulate the separation of thin, peelable films. The parameters of the cohesive zone model required for the simulation were derived from the force–displacement curves. In [12], the behavior of the adhesives between the aluminum layer and the adjacent layer of the composite film is investigated. The sealing seam formation is not considered in the numerical simulations in these publications. In [13], the T-peel test of welded, coated fabric films is simulated, considering the squeezed-out melt. However, the actual seam separation process was not considered. For the sake of simplicity, only the crack initiation stage was simulated. The influence of the sealing seam geometry on the separation behavior could, therefore, be demonstrated here by simulation.

This paper focuses on the modeling and simulation of the seal seam formation to prepare a method for the prediction of peeling behavior in complex seal areas and display local scale effects, which are a challenge for experimental representation.

Section 2 describes the material selection, the methodologies for determining strength, the sealing process, the parameter evaluation, and the fracture analysis. Section 3 outlines the modeling of the material with its individual layers, the fracture model, the geometry model, and the process model, as well as its implementation into the numerical simulation environment LS-DYNA. It also describes the execution of the numerical simulation and the comparison of the simulation results with the experimental findings. Section 5 provides an assessment and contextualization of the results, while Section 6 presents a summary of the outcomes achieved.

## 2. Materials and Methods

A common peelable PET-PE 12/50 µm composite was selected and used for the investigations. PET-PE composites are a popular packaging material due to their combination of the advantages of PET, such as high stability, strength, good barrier properties, and transparency, with the flexibility and good sealing properties of PE.

**Figure 1:**
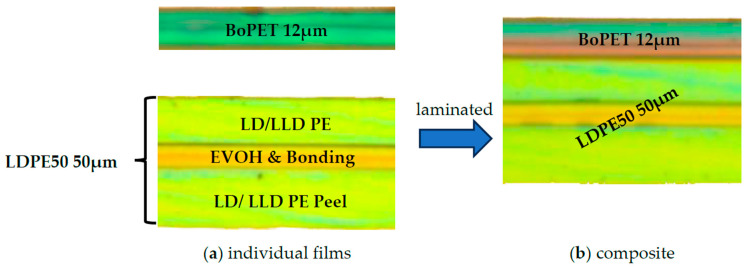
Peelable composite and its single layers: (**a**) single layer BoPET and single layer LDPE 50 were laminated to the PET-PE 12/50 composite (**b**).

The composite was laminated from the two individual extruded films, BoPET 12 and LDPE 50, as shown in Figure 1. The LDPE 50 film consists of three coextruded layers—an LD/LLD polyethylene layer, an adhesion promoter and EVOH layer, and an LD/LLD peelable polyethylene sealing layer. Due to the co-extrusion, these three layers are not available as individual films, and the LDPE 50 layer is, therefore, regarded as a homogenized polyethylene film. Therefore, a material characterization of the PET-PE 12/50 µm composite and its individual components, BoPET 12 and LDPE 50, was possible.

**Figure 2:**
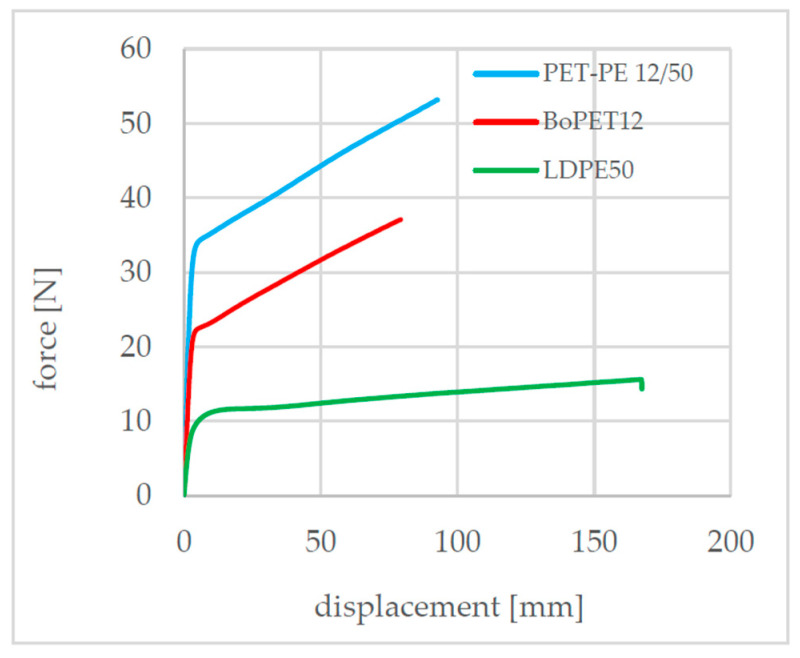
Experimental force–displacement curves from the tensile test.

To determine the mechanical behavior of the composite and the individual layers, tensile tests were carried out in the machine direction (MD) in accordance with DIN 527–3 with a clamping length of 100 mm and a test speed of 100 mm/min. The test specimen geometry was 15 mm wide and 150 mm long. This revealed different characteristic force–displacement curves for the PET-PE 12/50 composite and its individual layers, BoPET 12 and LDPE 50, as shown in Figure 2.

In the next step, heat contact sealing experiments were carried out with this material. The sealing parameters—sealing time (t**_S_**), sealing pressure (**p_S_**) and sealing temperature (T**_S_**)—were varied within the following ranges: the sealing temperature (T**_S_**) ranged from 110 °C to 150 °C, the sealing pressure (p**_S_**) ranged from 0.5 MPa to 6 MPa, and the sealing time (**t_S_**) ranged from 0.2 s to 0.75 s.

A labor sealer (HCT 3200, Willi Kopp e.K. Verpackungssysteme, Reichenbach an der Fils, Germany) was used with flat sealing jaws with a width of 5 mm. The seal samples were 30 mm wide and 100 mm long and were cut out in the machine direction (MD), and the sealing was carried out at a 90° angle to the machine direction.

**Figure 3:**
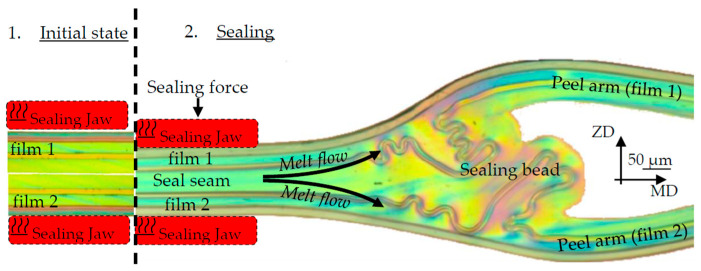
Sealing process and melt flow.

Microscope examinations (see Figure 3) show that during the sealing process, there is a melt flow, mainly in the MD, which leads to a sealing bead near the edge area. The unsealed area of the sealing samples remains as peel arms, which are clamped in the testing machine during the subsequent seam strength test.

The seam strength of the seal samples was then measured in the T-peel test in accordance with DIN 55529. The seam samples were initially stored at 23 °C and 50% humidity for 24 h. From the original 30 mm wide seal samples, 15 mm wide T-peel test samples were cut in the middle using a parallel blade in MD. In the T-peel test, one peel arm was clamped, and the other was pulled apart at a constant speed; the force and the displacement of the moving crosshead were recorded. The test parameters for the T-peel tests were as follows: a clamping length of 50 mm, a pre-force of 0.1 N, and a test speed of 100 mm/min under a test climate of 23 °C and 50% relative humidity.

**Figure 4:**
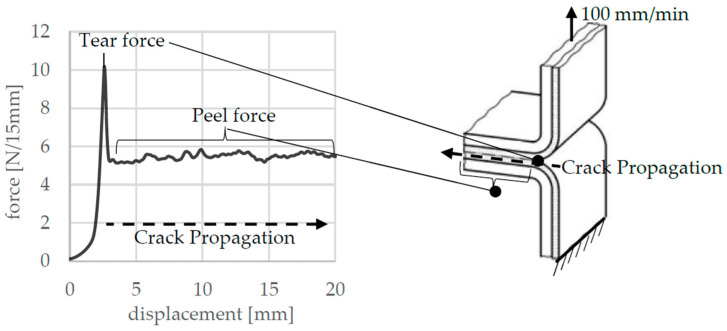
T-peel test and recorded force–displacement curve.

The test was used to determine the seam strength, the tearing force, and the fracture mechanism. Typical force–displacement curves, as shown in Figure 4, emerged with a force increase at the beginning and a maximum force, which is defined as the tearing force, and a subsequent plateau area in which the sample is peeled. This force is called peel force.

**Figure 5:**
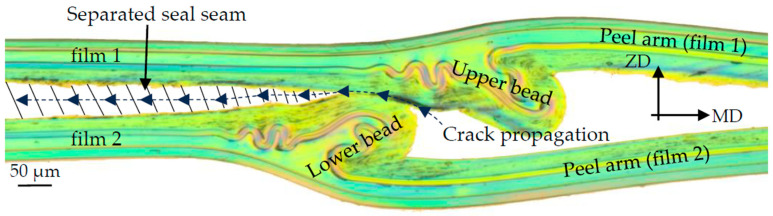
Fracture examination after the T-peel test.

To clarify the fracture behavior, a microscope examination of the seal sample was carried out after the T-peel test, which is shown in Figure 5.

The fracture analysis shows that the seal seam is separated in the middle between film 1 and film 2. The sealing bead is separated into an upper and lower sealing bead in the middle of the corridor formed by the EVOH layers of film 1 and film 2. From the fracture pattern, it is, therefore, possible to estimate the course of the crack as it occurs during the T-peel test. This fracture pattern occurred in all examined T-peel test results with fracture pattern type C according to DIN 55529.

## 3. Modeling

**Figure 6:**
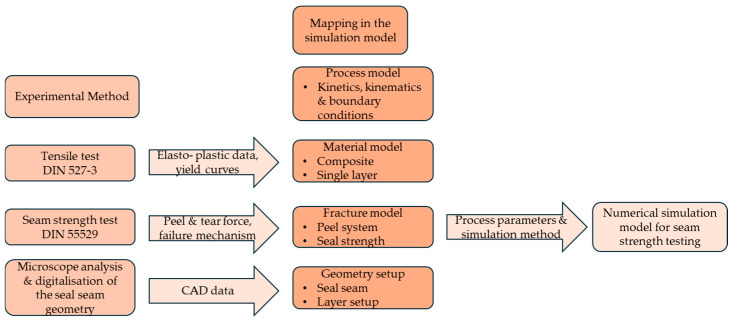
Schematic procedure for creating the simulation model from experimental methods and measured values.

To investigate the influence of the seal bead geometry on the seam opening forces and to identify the underlying mechanical effects, a numerical simulation model was developed. The model incorporates substitute parameters for the seam strength, which were derived from experimental methods and assumptions presented in Section 2. It includes process, material, fracture, and geometry models, as illustrated in Figure 6. While the focus of this study lies in analyzing the mechanical behavior and opening forces influenced by the seal bead geometry, the formation of the melt flow and bead geometry itself—determined by sealing parameters such as pressure, temperature, and time—was not part of the current investigation. Instead, the simulation assumes the existence of the seal seam and its geometry, as determined through experimental observations.

The process model depicts the essential boundary conditions and movements of the simulation and is derived from the analysis of the T-peel test used. Material parameters are obtained from the tensile tests from Figure 2, and the seal seam strength from the T-peel test. Microtome and microscopic analyses were used to determine the geometry of the seal seam and the seal seam formation. The seam strength analysis was simulated using the finite element software LS-DYNA (version 11.1, Livermore Software Technology Corporation, Livermore, CA, USA).

### 3.1. Material Model

In the material modeling process, the following assumptions were made: All materials were considered isotropic and homogeneous. Additionally, thermal effects during the opening process were neglected, as only the opening behavior in the cooled state was analyzed.

Engineering stress–strain curves (σ_e_-ε_e_) were derived from the measured force–displacement curves in tensile tests via

(1) $$\sigma_{e}=\frac{F}{A_{0}}$$

(2) $$\varepsilon_{e}=\frac{\Delta l}{l_{0}}.$$

σ_e_ in Equation (1) is the engineering stress resulting from the quotient of the measured force F and the initial cross-section $A_{0}$ of the sample. The engineering strain, ε_e_, in Equation (2) is calculated from the displacement of the traverse, Δl, and the initial length of the sample, $l_{0}$.

Since large deformations also occur in the real seam strength tests, the material behavior is also taken into account in the plastic range. Due to these large deformations, the simulation is non-linear and, therefore, the technical stress–strain curves must be converted into true stress–strain (σ_t_-ε_t_) curves, which are defined in Equations (3) and (4). Due to the measurement method, the conversion was carried out under the assumption of volume constancy [14].


σ_t_ = σ_e_ (1 + ε_e_) (3)

ε_t_ = ln (1 + ε_e_) (4)

However, the true strain–true stress curve of the material cannot directly be used for the simulation, as this could lead to numerical instabilities due to the smoothness of the curves. Therefore, the data must be fitted with the analytical G’sell–Jonas function [15]. The G’sell–Jonas function in Equation (5) represents the true stress $\sigma_{t}$ as a function of the true strain $\varepsilon_{t}$.

(5) $$\sigma_{t}\left(\varepsilon_{t}\right)=k\;*\;\left(1-e^{-({\frac{\varepsilon_{t}}{\varepsilon_{v}})}^{t}}\right)\;*\;e^{h{\varepsilon_{t}}^{u}}\;*\;\left(1-p\;*\;e^{-q\;*\;{\left(\varepsilon_{t}-\varepsilon_{D}\right)}^{2}}\right)$$

The curve parameters in the G’sell–Jonas function k, ε_v_, t, h, u, p, q, and ε_D_ [15,16] were determined using the Dual Annealing optimization algorithm based on the experimental data. As a result, a set of G’sell–Jonas curve parameters were found for every film (see Table 1). Both the individual films and the overall composite were optimized together, ensuring that both the behavior of the individual layers and the behavior of the overall composite under tensile load were correctly mapped.

The modulus of elasticity and the yield stress were then derived from the fitted stress–strain curves in accordance with DIN 527–3. The Poisson ratios and the density from Table 2 were taken from the literature [17,18,19,20], respectively. These data are listed in Table 2.

**Figure 7:**
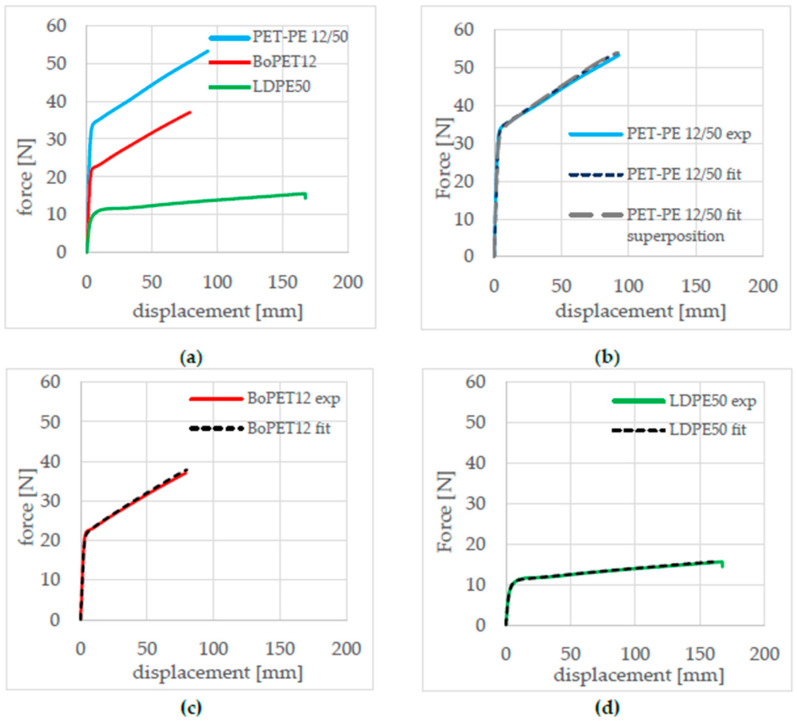
Results of the data fit. (**a**) Measured force–displacement curves from tensile test, (**b**) PET-PE 12/50 composite, (**c**) BoPET 12, (**d**) LDPE 50.

The determined elastoplastic material data and the plastic yield curves from the fitted G’sell–Jonas parameters were implemented in the material model *MAT_PIECEWISE_LINEAR_PLASTICITY (024 [21]) for the BoPET 12 and LDPE 50 layers. This material model makes it possible to implement the stress–strain values in tabular form and, thus, enables a very accurate representation of both elastic and plastic material behavior [22,23]. Figure 7b–d shows the optimization results of the individual films and the overall composite. Figure 7b shows that the material behavior of the overall composite PET-PE 12/50 can be superimposed on the material behavior of its individual films, BoPET 12 and LDPE 50. The comparison of the composite film’s optimization is based on three elements: the experimental curve of the entire composite (light blue line), its fitted curve (dark blue dashed line), and the superposition of the material properties of the individual layers, BoPET12 and LDPE50, as described in [6] (grey dashed curve).

### 3.2. Fracture Model

Four characteristic seal samples were selected from the statistical test plan with an expected low melt flow, a moderate melt flow, and two high melt flows to generate different melt beads and compare their behavior during seam opening. Their names and associated sealing parameters are listed in Table 3.

**Figure 8:**
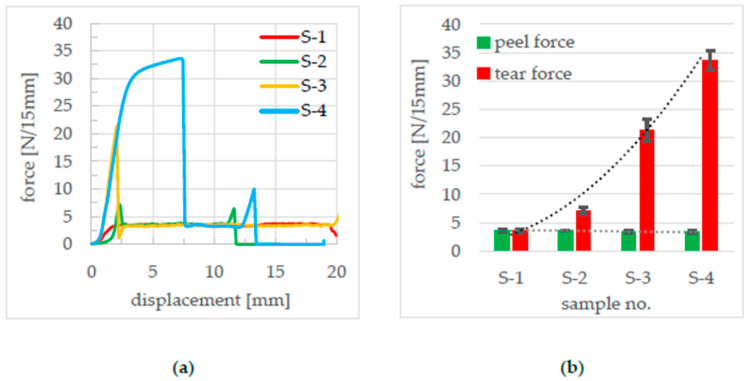
(**a**) Force–displacement curves T-peel test of the samples, (**b**) tear and peel forces.

From the 30 mm wide seal samples, 15 mm wide T-peel test samples were cut out in the machine direction and the center using a parallel blade. The T-peel test, in accordance with DIN 55529, was used to determine the seal seam strength, the tearing force, and the breaking behavior. Each test was repeated three times, and the average peel and tear forces were calculated from the measured force–displacement curves, as shown in Figure 8.

The force displacement curves in Figure 8 were different for each sealing parameter set. While a flat plateau area forms in S-1, an increase in the tearing force can be observed in samples S-2, S-3, and S-4. The peel force remains similar for all samples between 3.4 and 3.6 N/15 mm. The high tearing force in sample S-4 is due to a deformation of the peel arm before the sample peels. S-2 and S-4 show an increase in force at the end of the test, which can be explained by the tears at the other end of the seam.

The measured peel force is a measure of the breaking strength of the sealed seam, which can be used to derive the properties of the sealing layer in the simulation. Cohesive elements are suitable for modelling the crack propagation along a defined parting plane [11,12,24]. The material model *Cohesive_Mixed_Mode (MAT 138) was used in the simulation carried out here. In this model, the separation is elastic up to the failure limit. Load- and unloading-dependent damage development was not considered, as the load is constant during opening. As the seam opening behavior is simulated in the T-peel test in the later simulation model, no distinction is made between the normal and tangential parameters [24].

To determine the material parameters from the material model, an automatic parameter optimization was carried out with LS-OPT based on [11]. For this purpose, a T-peel test model was set up in LS-DYNA without taking the seal seam formation into account, and the parameters were optimized by parameter variation based on the experimentally determined peel force. As only one failure mode is considered, the mode mixity XMU remains 1. Table 4 provides an overview of the material parameters determined from the optimization for the cohesive zone model.

### 3.3. Geometry Model

**Figure 9:**
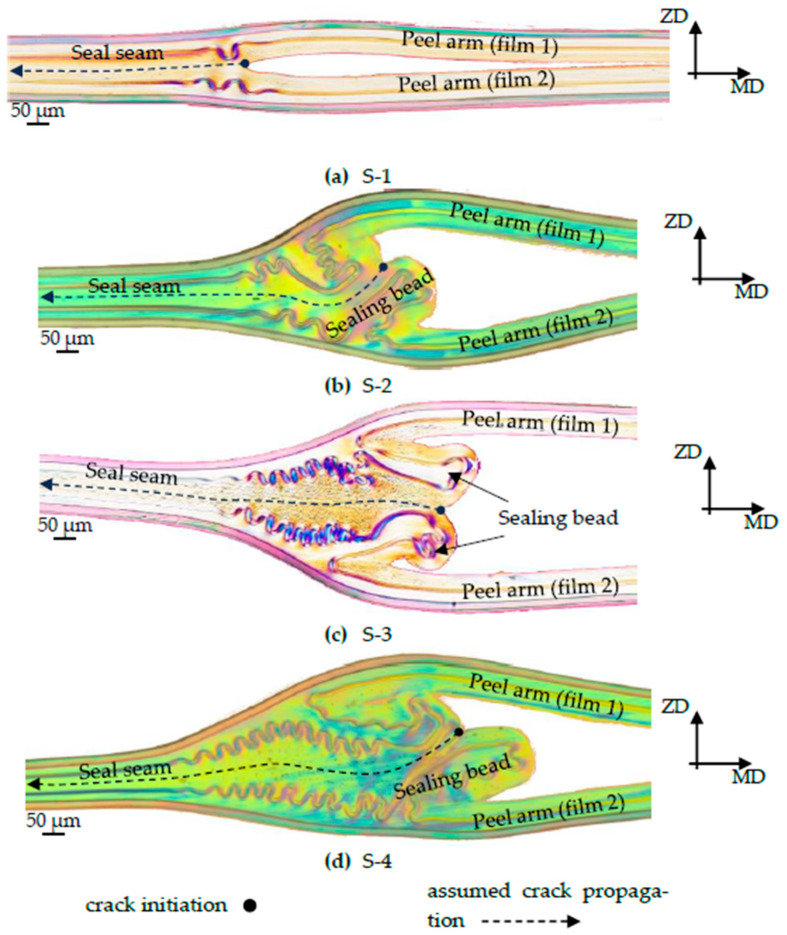
Sealing seam formations of sealing samples S-1 to S-4 (**a**–**d**) from Table 3 with assumed crack initiation and crack propagation.

To determine the seal seam formation of the four samples, microscopic images were made from microtome sections prepared from the side remnants of the T-peel test samples. The geometries of the seal seams were visualized, scaled, and are shown in Figure 9.

In samples S-2 to S-4, very pronounced sealing beads can be observed in the sealing edge area. This is due to the higher sealing temperatures on the one hand, and the higher sealing pressure on the other [4,25,26]. A remarkable feature is the double notch on S-3 and the almost twice-as-large sealing bead on S-4. The former is probably due to the longer sealing time, and the latter to the higher sealing pressure.

Using the spline geometry function from a CAD program such as SolidWorks, the seal seam formation could be reproduced in detail with its contours and beads. To the right of the seal seam, the bead merges into the peel arms, and to the left, the seal seam folding merges into the seal seam, in which the foils are welded together. Although no mesh sensitivity analysis was conducted, the chosen element sizes of 0.005 mm (longitudinal and vertical directions) and 0.01 mm (thickness direction) represented a fine discretization [27], ensuring that the stress and strain distributions were captured with high accuracy, as required for modeling the seal seam formation and crack propagation behavior. To save calculation time, only one element row was implemented in the thickness direction.

**Figure 10:**
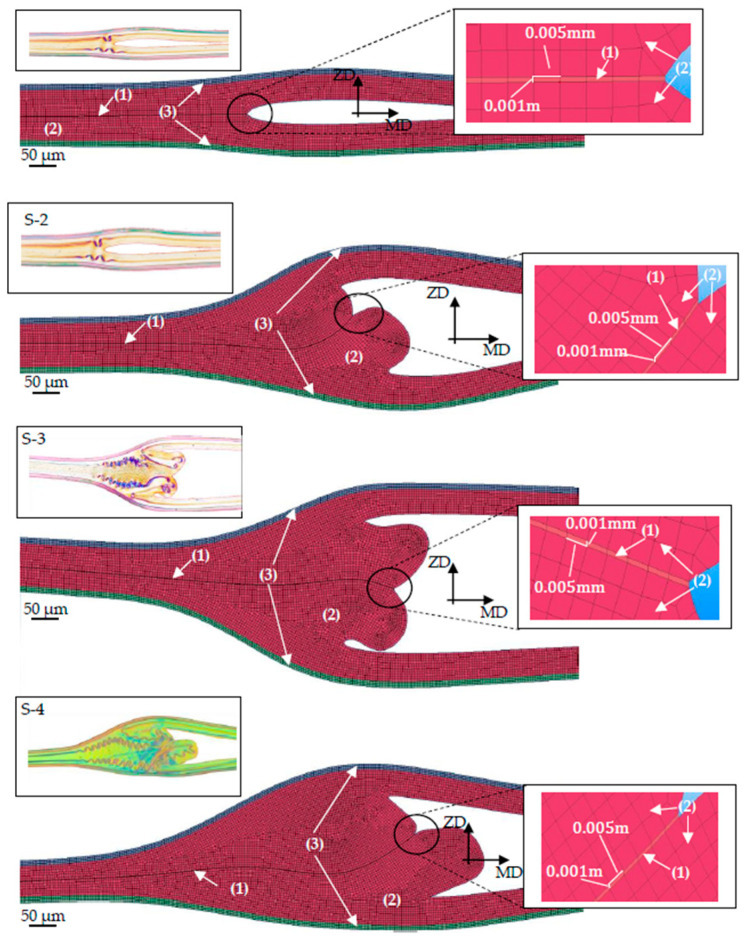
Meshed geometries of the seal seam bead and formation for S-1 to S-4. (1) Cohesive layer, (2) LDPE 50, (3) BoPET 12.

Based on the fracture behavior observed in Figure 5, the crack initiation and propagation mechanism were incorporated into the numerical model, as shown in Figure 9 and Figure 10, using cohesive elements, with element sizes of 0.005 mm in the longitudinal direction and 0.001 mm in the vertical direction. The assumed crack initiation point was located in the notch between the two films, and the cohesive elements described the crack propagation along the predefined parting plane. The geometry of the seal seam, including the bead and the cohesive zone, was discretized as shown in Figure 10.

The cohesive elements were connected to the LDPE 50 elements via shared nodes, as were the elements between the LDPE 50 and BoPET 12 layers. This modeling approach ensures that no friction or relative displacement occurs between the elements.

### 3.4. Process Model

**Figure 11:**
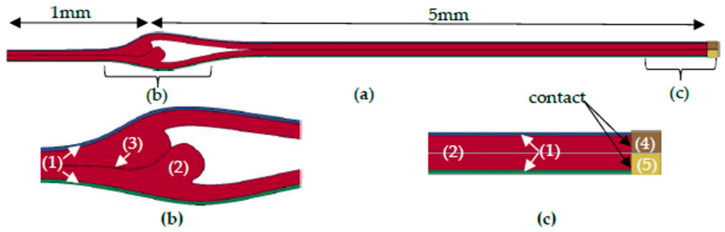
Simulation model of the T-peel test, taking into account the seal seam formation. (**a**) Complete model, (**b**) detailed view of the seal seam formation, (**c**) detailed view of the peel arms and clamps (1) BoPET 12, (2) LDPE 50, (3) cohesive layer, (4) top clamp, (5) bottom clamp.

In the next step, a finite element simulation model of the T-peel test was implemented for samples S-1 to S-4, considering the seal seam formation, as shown in Figure 11. The seam opening process in the numerical simulation model based on the T-peel test and the simulation was performed quasi-statically without considering dynamic or cyclic effects, as the real T-peel test process is also carried out continuously until the seam fails. With geometric dimensions of 1 mm in length of the seal seam and 5 mm for the peel arms, the proportions of the experimental T-peel test samples were considered in the simulation model. The simulation was carried out with the finite element software LS-DYNA® R11.1, double precision, and was solved using a nonlinear implicit solver. All material and process variables used were implemented in the unit system kN-mm-ms.

Contact boundary conditions were implemented to extract the reaction forces between the material and the clamps. These reaction forces were subsequently compared to the experimentally determined forces to evaluate the simulation accuracy. In the simulation models, a contact *TIED_SURFACE_TO_SURFACE_OFFSET was used. The contact interaction between the clamps and the film is assumed to be perfectly bonded.

For the material description and the moving of the clamps, the *MAT_RIGID (Mat 20) was used [20,21]. The material properties assigned to the clamps had a density of 7.86 × 10^−6^ kg/mm^3^, a Young’s modulus of 210.0 kN/mm^2^, and a Poisson’s ratio of 0.3.

The film was preconditioned by simulating the folding and clamping process to replicate the initial state of the experimental samples. The upper clamp moves in the y-direction at a constant velocity of 0.0016 mm/ms, which corresponds to an experimental speed of 100 mm/min. The lower clamp, on the other hand, was fixed in all degrees of freedom during the opening phase of the T-peel test.

**Figure 12:**
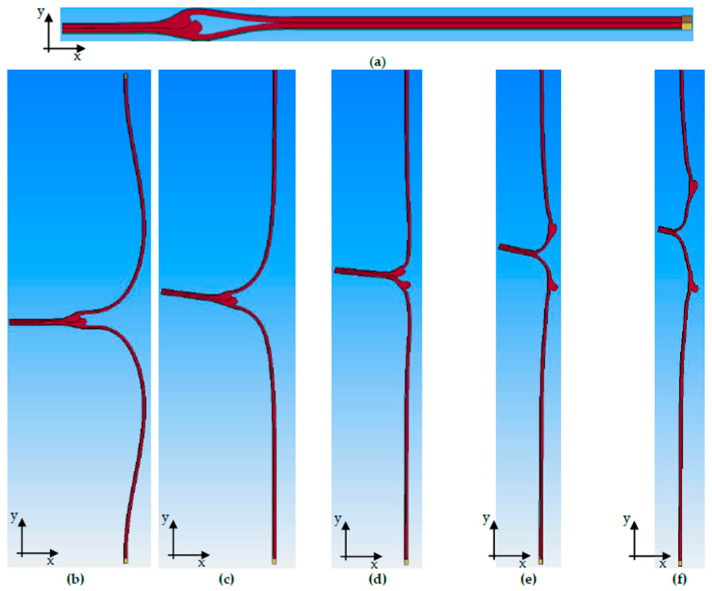
Simulation procedure: (**a**) initial state (peel arms folded), (**b**) opening of the peel arms to vertical position, (**c**) continuous movement of the upper clamp in y-direction, (**d**) first crack in the seal bead, (**e**) crack progress in the seal seam, (**f**) end of simulation.

Thus, the T-peel test sample was separated along the cohesive zone. The simulation process of the seam opening process, taking into account the seal seam formation, is shown for S-2 in Figure 12. The simulation was performed analogously for the other geometries S-1, S-3, and S-4. It should be noted that the y-direction of the simulation corresponds to the ZD in the experimental investigations.

## 4. Results

On the one hand, the stress states in the sealing seam, in the sealing bead, and in the cohesive elements prior to crack initiation were examined for evaluation. Furthermore, the simulation-generated force–displacement curves were compared with the experimentally measured force–displacement curves and used to evaluate the simulation quality and plausibility of the simulation method shown here. In addition, parameter studies were carried out via simulation by varying the seal seam strength to investigate the influence of the seal seam strength on the opening behavior.

**Figure 13:**
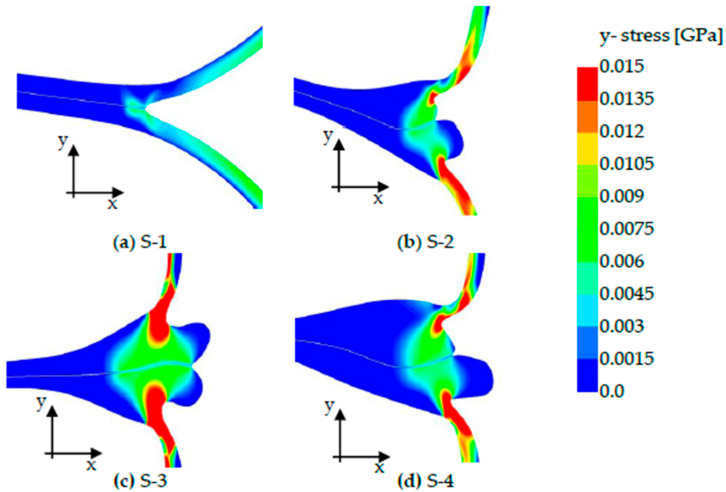
Stress application in the y-direction in samples S-1 to S-4. The subfigures (**a**–**d**) show the stress distribution in the sealing seams before failure.

The comparison of the stress distribution of the y-stress in the different seal seam formations of samples S-1 to S-4 (see Figure 13) shows that the greatest stress occurs in S-2 to S-4; however, they do not act directly in the parting plane. As described in [4] the peel arm constricts at the transition to the sealing bead. Here, the greatest stresses occur locally. This is due to the thin peel arm compared to the wide sealing seam. With S-1, the greatest tension acts directly in the sealing seam. In addition, it can be concluded from the peel arms that sample S-1 is bent open more, as already described in [28]. The same tension is applied here to the sealing seam, even though the peel arms are not yet aligned vertically with the sealing seam.

The size of the area into which the force is applied plays a special role here. Depending on how large the seal seam formation is, this leads to a different distribution of stress in the sealing seam and, thus, to higher bearable forces before the crack. Measuring the length of the cohesive elements of the sealing seam that are affected by the stress introduction in Figure 13 yielded the values listed in Table 5. This shows that the stress application in samples S-3 and S-4 acts on a larger affected length.

**Figure 14:**
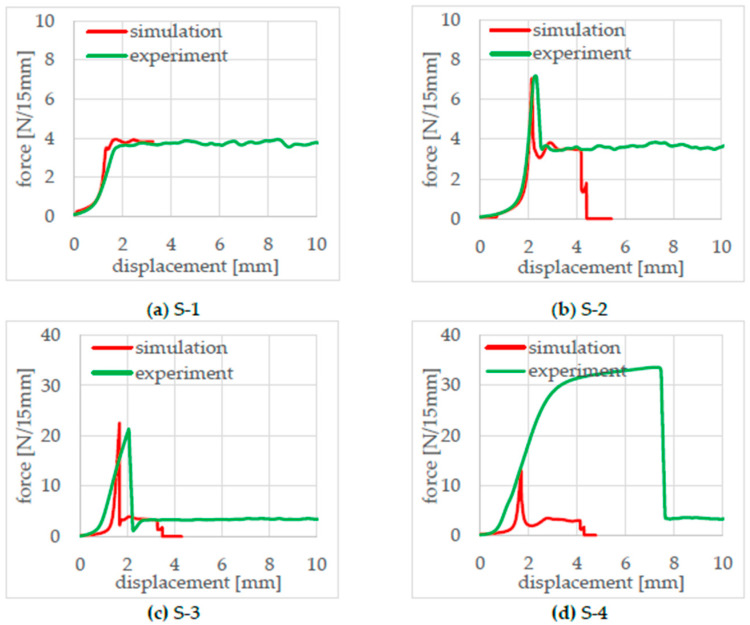
Comparison of the simulation force–displacement curves with the experimental ones of specimens S-1 to S-4 (**a**–**d**).

In addition, after the simulation process, the simulated and experimentally generated force–displacement curves were compared with each other. For this purpose, the reaction force and the displacement path in the y-direction of the upper clamp were extracted from d3plot files and plotted on top of each other. The simulated force–displacement curve of the seam strength analysis was then compared with the experimental measurements, as shown in Figure 14.

As shown in Figure 14a,b, there is very good agreement between the experimental and simulation results for samples S-1 and S-2. This is because the failure mechanism is a pure peel failure along the seal seam, which can be accurately modeled using cohesive elements in the simulation. However, the failure mechanism for S-3 and S-4 is more complex. In Figure 14c, the force increase for S-3 in the simulation is steeper than in the experiment, while the initiation force and the peel force show good agreement. In contrast, Figure 14d highlights the significantly more complex failure mechanism observed in the experiment for sample S-4. Before failure, material softening occurs, leading to fracture only after greater deformation in the seal bead and in the peel arms. A simplified assumption of crack initiation, crack propagation, and material properties within the seal bead is no longer valid for this case. The explanations for this phenomenon are described in greater detail in Section 5.

**Figure 15:**
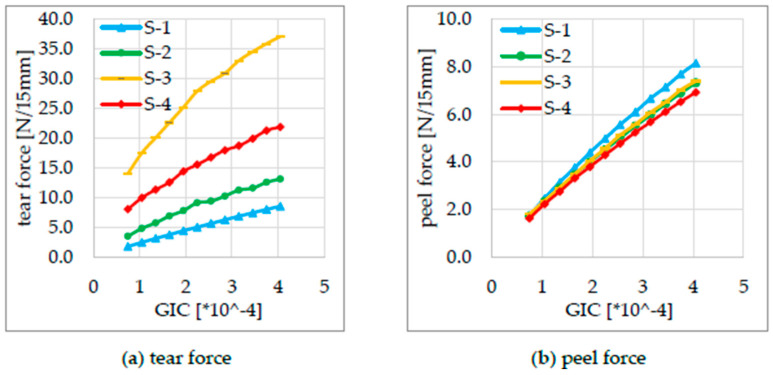
Effects of the seal seam strength on the (**a**) tear force and (**b**) the peel force, taking into account the seal seam formation for specimens S-1 to S-4.

To verify the geometric influence of the seal seam formation, simulation-based parameter studies were carried out for all samples in which the seal seam strength of the cohesive elements was artificially varied, i.e., both strengthened and softened. For this purpose, the factor GIC and its dependent variables were changed in the cohesive zone model [20], and the simulation was carried out and evaluated. The fracture always took place in the specified fracture zone of the cohesive materials. As shown in Figure 15a, the tear force varies significantly among samples S-1 to S-4. The highest rupture force is observed for S-3 due to its seal bead geometry, as it has the largest effective length over which the stress is distributed, followed by S-4, S-2, and S-1, as described in Table 5.

In contrast to the tear force, the peel force remains consistently lower because the stress is introduced only into the edge region of the seal seam, rather than being distributed across a larger line segment of the seal bead. This, combined with the thinning of the seal seam in S-2, S-3, and S-4 compared to S-1, leads to a lower reaction force due to the reduced cross-sectional area of the film in the seal seam region.

## 5. Discussion

A pronounced melt flow leads to a larger parting surface into which the stress is introduced, which results in greater tear forces (greater crack initiation forces) to be applied. The lengths shown in Table 5, in which the stress is applied, correlate with the simulation-calculated size of the crack initiation force. The greatest tearing force occurred at S-3, lower forces at S-4 and S-2, and the lowest tearing force at S-1. In addition, it can be observed for S-2 to S-4 that the greatest stress does not act directly in the sealing seam, but at the transition between the peel arm and the sealing bead, as described in [2]. At S-1, it is noteworthy that a similarly high stress is present in the seal seam strength, although the peel arms are not yet aligned perpendicular to the seal seam; see Figure 13a. It can, therefore, be assumed that the tension is caused more by bending than by peeling, as already described in [28].

**Figure 16:**
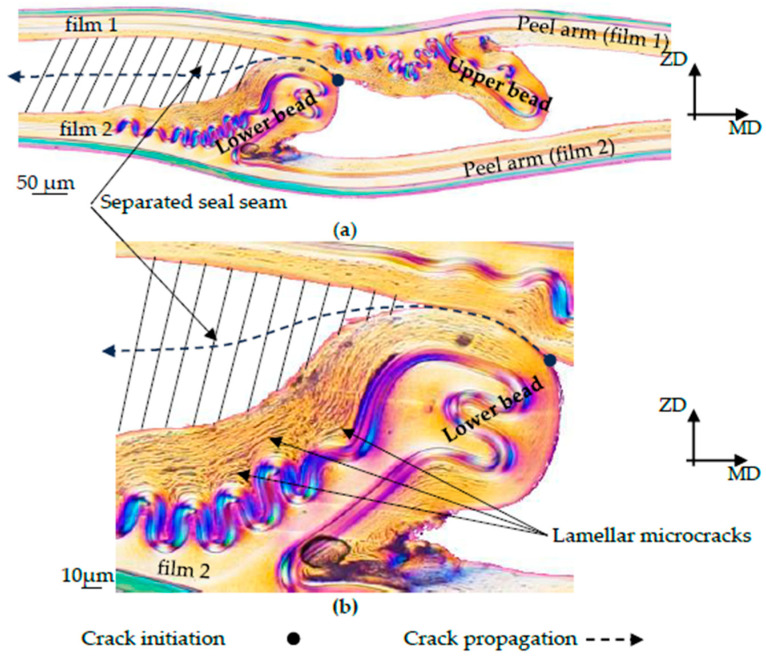
Fracture pattern of sample S-3. (**a**) Crack pattern through the sealing seam, (**b**) detailed view of the lower sealing bead with lamellar microcracks.

Samples S-1 and S-2 show good agreement with the experimental results, both in terms of the increase in force, the tearing force, and the subsequent peel force. Although sample S-3 shows good agreement with the tearing force and the subsequent peel force, the increase in force to the tearing force differs between the simulation and experiment. The fracture, as shown in Figure 16, occurs along the assumed fracture plane. Upon closer inspection, lamellar microcracks can be observed in the lower part of the bead (see Figure 16b) within the LDPE 50 area. It is conceivable that material softening first occurs in the bead outside the sealing area, particularly in the LDPE 50 layer, before the crack is initiated in the sealing plane and the seam separates.

**Figure 17:**
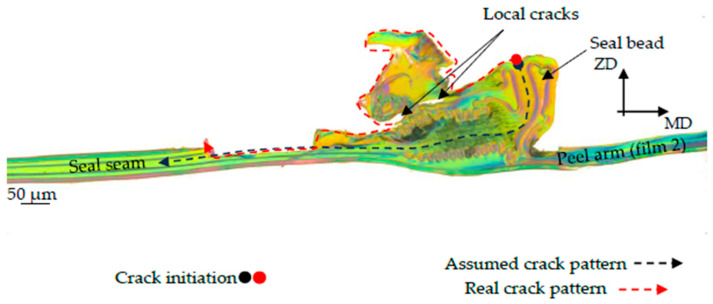
Fracture pattern of sample S-4.

In sample S-4, the simulation results deviate from the experimentally measured curve. During the T-peel test experiments, a plastic deformation of the peel arm was observed in sample S-4 before peeling occurred. In Figure 9d, sample S-4 shows that the EVOH layers touch each other in the sealing bead. It is conceivable that the peel layer has been completely displaced in this area, and a tightly sealed seam has formed locally, which explains the very strong increase in force. However, the analysis of the fracture pattern of sample S-4 in Figure 17 indicates that the assumed fracture pattern from Figure 9d is not correct. Although Figure 17 only shows the lower half of the fracture pattern, it is still possible to draw conclusions about the crack pattern. Instead of a separation between the EVOH layers of film 1 and film 2, the sealing bead above the EVOH layer of film 1 is torn open. This results in an undefined crack propagation through the squeezed-out LDPE 50 material. This suggests that the crack does not occur at the interface of the EVOH layers but rather propagates through the LDPE 50 layer. In addition, it can be assumed that plastic deformation also occurs in the sealing bead area due to local cracks being visible. These are much more pronounced here than with S-3. Only then does a peel effect occur in the expected parting plane, which explains the peel force in the experiment.

**Figure 18:**
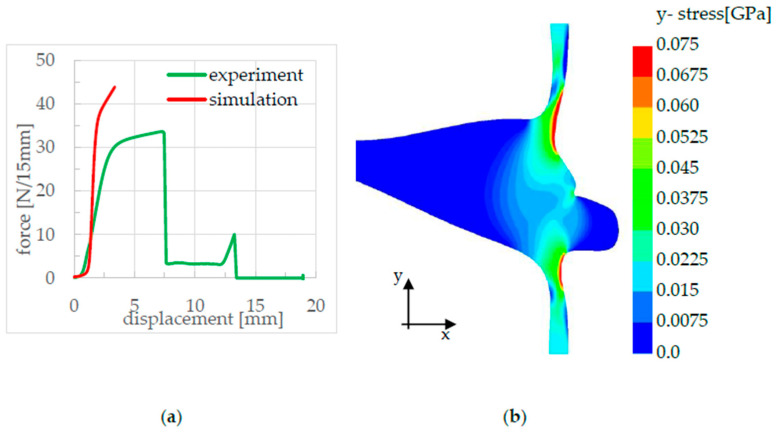
(**a**) Comparison of force–displacement curves between experiment and simulation from S-4, (**b**) y-stress distribution at 3 mm displacement.

In addition, after peeling, there is a tear through the sealing seam, and no further peeling effect occurs. The sealing seam may be thinned locally due to the high sealing pressure and the previous deformation, so that the local strength of the sealing seam exceeds the material strength of film 1, which is why a tear occurs through film 1 and not, as expected, the peeling of the sealing seam. As described in [29], the peeling effect is created by polybutene 1 blends in the sealing layer. These small particles ensure local disconnectedness between the sealing layers of film 1 and film 2, which enables easy opening. The fact that other peeling effects occur in samples S-3 and S-4 can be explained, on the one hand, by the enlarged parting plane, as shown in the simulation, which requires a higher application force, and on the other hand, by the fact that the polybutene 1 particles are swirled by the large melt flow and, therefore, there is no longer a defined peel layer, so that, as seen in S-4, there are local solidifications, as shown in Figure 9d. Since the fracture evaluation at S-4 showed material fracture through the sealing bead, the simulation of S-4 without the cohesive layer was repeated, the stress distribution was analyzed, and the force–displacement curves were compared with the experiment in Figure 18.

The comparison of the force–displacement curve between the experiment and simulation shows that without the cohesive layer, the simulation qualitatively falls within the force range of the experiment. However, the force increases, force curves, and maximum forces differ from each other. The analysis of the stress distribution at 3 mm displacement shows that the bearable stresses are three times as high as in the model with a cohesive layer and that the maximum stresses occur at the transition between the peel arm and the sealing bead, as already shown in [4]. Accordingly, failure (tearing) of the peel arm at the sealing bead would also be expected, as shown by the stress state. However, the analysis of the fracture showed that there is a material fracture in the sealing bead. In [4], however, ultrasonic sealing seams were investigated, which fail, in particular, at the morphological boundaries between melted and cold material areas at the transition between the peel arm and the sealing bead. It was concluded that the simulation can be used to estimate the failure of ultrasonic seams. The failure mechanism of sealed products produced with heat contact sealing, where no classic peel effect occurs, is more complex to model. It can be assumed that the material behavior in the sealing bead leads to material changes due to the thermomechanical stress during sealing (heating–stretching–cooling), which differs fundamentally from the material behavior in the simple tensile test. In the experiment, a softening was observed that does not occur in the simulation. Morphological changes in the material are, therefore, very likely. The stress distribution is qualitatively correct, but the fact that the failure takes place in the middle of the bead suggests that there is a different material behavior here, which leads to crack initiation even at lower stresses. In addition, the material in the T-peel test is also predominantly stressed in ZD, which does not emerge from tensile tests in MD.

**Figure 19:**
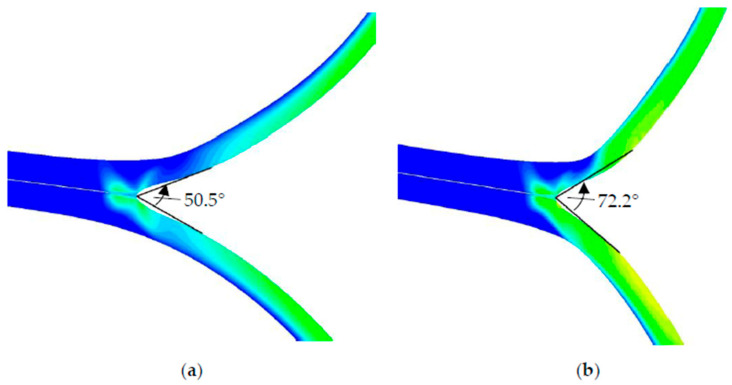
Opening angle of sample S-1 at (**a**) GIC = 1.7*10^−4^ and (**b**) GIC = 4.0*10^−4^ before the crack.

The simulation study varying the seal seam strength by variation of GIC and the variables dependent on it shows that the geometric influence on the tearing force is independent of the seal seam strength. The qualitative differences in the tearing force remain for all investigated seal seam formations S-1 to S-4, according to which S-3 has the highest tearing force, followed by S-4, S-2, and S-1. The situation is different for the peel force. Here, a higher peel force is calculated with a higher seal seam strength at S-1. While this is relatively similar for S-2 and S-3, it drops significantly for S-4. On the one hand, the increasing seal seam strength at S-1 ensures that the seam is pulled open more and bent open less, as the peel arms are at a steeper angle to the seal seam, as shown in Figure 19.

The seal seam is also less thinned out than with S-2 to S-4, resulting in greater reaction forces in the peel area, as the PE layers in the seal seam are even thicker. This effect of the slightly higher peel force with S-1 compared to S-3 and S-4 could also be measured in the experiments (see Figure 4).

## 6. Conclusions

The primary aim of this study was to analyze the influence of seal bead geometry on seam opening forces and to identify the underlying mechanical effects. It was demonstrated that the seal bead formation has a significant impact on the opening behavior, which could not be determined through experimental seam strength tests alone. Relevant parameters were derived from material, fracture, and geometry analyses and incorporated into a numerical simulation model of the T-peel test. Parameter studies revealed that a pronounced seal bead formation increases the parting surface, requiring greater tearing forces to peel the seal seam.

The presented method reliably predicts seam strength while considering the seal bead formation for fracture type C according to DIN 55529, provided that the fracture occurs along the seam and plastic deformation outside the seam remains minimal. This justifies the use of a cohesive element formulation with elastic deformation up to failure. However, for samples S-3 and S-4, atypical force–displacement curves were observed due to local plastic deformation or crack propagation outside the sealing layer.

A deeper understanding of the material behavior and local effects caused by material changes in the seal bead, as well as the behavior in the ZD direction (as observed in the T-peel test), is necessary to model more complex mechanisms. Future studies should investigate the formation of the melt flow and bead geometry in greater detail, including the influence of temperature, pressure, and sealing time on the molten material’s flow behavior. Coupling thermal and flow models could provide deeper insights into the relationship between process parameters, melt flow, and bead geometry.

The results of this study provide valuable insights into the mechanical behavior of the seal seams with varying bead geometries. These findings can be applied to optimize sealing parameters in industrial processes, ensuring a balance between seam strength and ease of opening. 

## Figures and Tables

**Table 1:** Optimized parameters of the G’sell–Jonas material model.

	k	ε_v_	t	h	u	p	q	ε_D_
BoPET 12	0.11	0.016	1.38	1.95	0.95	9.98	12.38	1.29
LDPE 50	0.016	0.0201	1.05	1.36	1.03	0.22	9 × 10^−6^	9.83
PET-PE 12/50	0.034	0.016	1.31	1.76	1.04	20.05	27.29	0.91

**Table 2:** Elastoplastic material data.

	E [kN/mm^2^]	σ_y_ [kN/mm^2^]	ν [-]	ρ [kN/mm^3^]
BoPET 12	4.25	0.021	0.4	1.38 × 10^−6^
LDPE 50	0.61	0.0015	0.42	9.97 × 10^−7^
PET-PE 12/50	1.14	0.0028	0.4	1.07 × 10^−6^

**Table 3:** Selected seal samples.

Sample Name	p_s_ [MPa]	t_s_ [s]	T_s_ [°C]
S-1	1	0.75	130
S-2	3	0.5	150
S-3	5	0.75	150
S-4	6	0.5	150

**Table 4:** Optimized parameters of the cohesive zone model.

Sample	Peel Force [N/15 mm]	EN	GIC	T	UND
S-1	3.572	10	1.7 × 10^−4^	0.00583	0.0583
S-2	3.523	10	1.68 × 10^−4^	0.00579	0.0579
S-3	3.903	10	1.86 × 10^−4^	0.00609	0.0609
S-4	3.4	10	1.62 × 10^−4^	0.00569	0.0569

**Table 5:** Affected length of stress introduction.

Sample	Affected Length [mm]
S-1	0.0625
S-2	0.0981
S-3	0.223
S-4	0.125

## Data Availability

The raw data supporting the conclusions of this article will be made available by the authors on request.

## Abbreviations

The following abbreviations are used in this manuscript:

PET	Polyethylene terephthalate
PE	Polyethylene
LDPE	Low-density polyethylene
BoPET 12	Biaxially oriented polyethylene terephthalate film with 12 µm thickness
LDPE 50	Low-density polyethylene film with 50 µm thickness
EVOH	Ethylene vinyl alcohol copolymer
t_S_	Sealing time
p_S_	Sealing pressure
T_S_	Sealing temperature
MD	Machine direction
ZD	z-direction (thickness direction)
σ_e_	Engineering stress
*F*	Force
*A*_0_	Initial cross-sectional area
ε_e_	Engineering strain
*l*_0_	Initial length
σ_t_	True stress
ε_t_	True strain
$k,\;\varepsilon_{V},\;t,\;h,\;u,p,q,\varepsilon_{D}$	Material parameter of the G’sell–Jonas model
E	Young’s module
σ_y_	Yield stress
ν	Poisson’s ratio
ρ	Density
S-1	Seal sample 1
S-2	Seal sample 2
S-3	Seal sample 3
S-4	Seal sample 4
MAT 138	COHESIVE_MIXED_MODE material model in LS-DYNA
EN	Stiffness normal to the plane of the cohesive element
GIC	Energy release rate for mode I (normal to the plane)
T	Peak traction in normal direction
UND	Ultimate displacement in normal direction
XMU	Exponent of the mixed-mode criteria
MAT 24	PIECEWISE_LINEAR_PLASTICITY material model in LS-DYNA
CAD	Computer-aided design
